# *Prostate Tumor Overexpressed 1 (PTOV1)* Is a Novel Prognostic Marker for Nasopharyngeal Carcinoma Progression and Poor Survival Outcomes

**DOI:** 10.1371/journal.pone.0136448

**Published:** 2015-08-25

**Authors:** Qi Yang, Huanxin Lin, Shu Wu, Fangyong Lei, Xi Zhu, Libing Song, Minghuang Hong, Ling Guo

**Affiliations:** 1 Sun Yat-sen University Cancer Center, State Key Laboratory of Oncology in South China, Collaborative Innovation Center for Cancer Medicine, Guangzhou, 510060, P.R. China; 2 Department of Nasopharyngeal Carcinoma, Sun Yat-sen University Cancer Center, Guangzhou, 510060, P.R. China; 3 Department of Radiation Oncology, Sun Yat-sen University Cancer Center, Guangzhou, 510060, P.R. China; 4 Department of Experimental Research, Sun Yat-sen University Cancer Center, Guangzhou 510060, P.R. China; 5 Department of Clinical Trials Center, Sun Yat-sen University Cancer Center, Guangzhou, 510060, PR China; The University of North Carolina at Chapel Hill, UNITED STATES

## Abstract

**Background:**

Prostate tumor overexpressed 1 (PTOV1) has been reported to contribute to increased cancer proliferation. However, the clinical significance of PTOV1 in the development and progression of nasopharyngeal carcinoma (NPC) is unclear. Our study aimed to investigate the expression pattern of PTOV1 in NPC and its correlation with clinicopathological features of patients.

**Methods:**

Western blotting and real-time PCR were conducted to examine PTOV1 expression levels in NPC cell lines and biopsy tissues compared with normal controls. Immunohistochemistry (IHC) was performed to analyze PTOV1 protein expression in paraffin-embedded tissues from 123 patients. Statistical analyses were applied to evaluate the clinical significance of PTOV1 expression.

**Results:**

PTOV1 mRNA and protein levels were upregulated in NPC cell lines and clinical samples. IHC analyses showed that PTOV1 was highly expressed in 68 (55.3%) of 123 NPC specimens. Statistical analysis revealed that PTOV1 expression was significantly correlated with clinical stage (P < 0.001), T classification (P = 0.042) and N classification (P = 0.001). Patients with a higher PTOV1 expression had shorter overall survival compared with those with a lower PTOV1 expression level, especially in lower N stage patients. Multivariate analyses suggested that PTOV1 expression was an independent prognostic marker for survival in NPC patients.

**Conclusions:**

Our data demonstrated that PTOV1 overexpression is associated with poor survival outcomes of NPC patients, especially in N0-1 patients. Hence, PTOV1 may help to detect early lymph node metastasis of NPC patients and serve as an independent prognostic biomarker for human NPC.

## Introduction

Nasopharyngeal carcinoma (NPC) is an endemic head and neck tumor in southern China, of uncertain etiology [1, 2]. On account of its abundant supply of regional lymphatic vessels, NPC has a tendency to metastasize initially to the regional lymph nodes [3]. Roughly 75% of NPC patients have regional lymph node involvement at diagnosis [4]. Moreover, patients with advanced N stage initially have a higher frequency of distant metastases, which is the pivotal contributor to NPC mortality [4, 5]. A retrospective study demonstrated that lymph node involvement is the most significant determinant factor of patient survival outcomes in NPC [6]. Although radiotherapy remains the standard treatment for NPC, the outcome for locoregional advanced cases is disappointing [3, 7]. Therefore, the discovery of novel biomarkers associated with the diagnosis and progression of NPC would be of great value in identifying high-risk patients who may benefit from more aggressive clinical intervention.

PTOV1 comprises two highly homologous domains arranged in tandem, and is encoded by a 12-exon gene localized on chromosome 19q13.3 ± 13.4. This gene was originally identified by a differential display search for molecular markers of progression in prostate cancer (PCa) [8]. PTOV1 contributes to the proliferative status of prostate tumor cells and plays an ancillary role in the nuclear entry and mitogenic activity of the lipid raft protein flotillin-1 [9, 10]. Subsequent studies explored PTOV1 immunoreactivity in diverse PCa-related cases and found that PTOV1 expression increased from normal-looking epithelium (NEp) of the transition zone (TZ) through atypical adenomatous hyperplasia (AAH) and high-grade prostatic intraepithelial neoplasia (HGPIN) to PCa, suggesting its role in prostatic carcinogenesis [11–13]. Fernández S *et al*. used microarray analysis to show that PTOV1 may serve as a new marker of aggressive diseases in 12 carcinomas, especially in high-grade tumors [14]. Furthermore, PTOV1 is considered one of the testosterone-induced atherogenic genes, which are involved in vascular smooth muscle cells (VSMCs) proliferation [15]. Recently, PTOV1 was shown to cooperate with Zyxin to reduce retinoic acid (RA) sensitivity, which is critical for cancer therapy with retinoids [16, 17]. Accumulating evidence has revealed that PTOV1 plays a vital role in carcinogenesis and tumor progression. However, the clinical significance of PTOV1 in NPC remains unknown.

In this study, we found that PTOV1 was upregulated in NPC cell lines and clinical samples at both the protein and mRNA levels. Moreover, PTOV1 expression levels were correlated statistically with clinical stage, T classification, N classification and vital status of NPC patients. Multivariate analysis revealed that PTOV1 was an independent prognostic marker for patients' survival in NPC. Taken together, our results suggest that PTOV1 may help to detect early lymph node metastasis of NPC patients and serve as a promising prognostic biomarker in human NPC.

## Materials and Methods

### Ethic statement

For the use of the clinical materials for research purposes, prior written informed consents from all patients and approval from the Institute Research Ethics Committee of Sun Yat-sen University Cancer Center were obtained.

### Cell lines

Normal nasopharyngeal epithelial cell line (NPEC) was cultured in Keratinocyte serum-free medium (Invitrogen, Carlsbad, CA, USA) supplemented with epithelial growth factor, bovine pituitary extract and antibiotics (120 μg/ml streptomycin and 120 μg/ml penicillin) [18]. Human NPC cell lines, including HK1, CNE1, CNE2, SUNE1, Hone1, 5-8F and 6-10B were purchased from ATCC and grown in RPMI 1640 (Invitrogen) supplemented with 10% FBS (HyClone, Logan, UT), 100μg/μL streptomycin, and 100μg/μL penicillin.

### Patient information and tissue specimens

Freshly frozen tissue samples of eight nasopharyngeal carcinoma biopsies and six noncancerous nasopharyngeal biopsies were obtained under fiberoptic nasopharyngoscopy from the Department of Nasopharyngeal Carcinoma, Sun Yat-sen University Cancer Center. A total of 123 paraffin-embedded NPC samples, which were histologically and clinically diagnosed between 2007 and 2010 at the Sun Yat-sen University Cancer Center, were also included in this study [19]. All NPC cases were classified as undifferentiated Nonkeratinizing Carcinoma (WHO III). The disease stages of the patients were reclassified according to the seventh American Joint Committee on Cancer TNM staging manual [20]. Clinical information of the samples is summarized in Table 1. The follow-up time of the study cohort ranged from 14 to 72 months, and the median follow-up time was 55 months.

**Table 1:** Clinicopathological characteristics of patient samples and expression of PTOV1 in NPC and correlation between PTOV1 expression and clinicopathological characteristics of NPC patients.

Characteristics	Total (n = 123)	PTOV1 Low expression (%)	PTOV1 High expression (%)	chi-square test P value
Gender				0.228
Male	83	34(41.0)	49(59.0)	
Female	40	21(52.5)	19(47.5)	
Age(y)				0.073
≤44	65	34(52.3)	31(47.7)	
>44	58	21(36.2)	37(63.8)	
Clinical stage				<0.001
II	8	8(100.0)	0(0.0)	
III	65	33(50.8)	32(49.2)	
IV	50	14(28.0)	36(72.0)	
T stage				0.042
T_1_	1	1(100.0)	0(0.0)	
T_2_	26	15(57.7)	11(42.3)	
T_3_	67	32(47.8)	35(52.2)	
T_4_	29	7(24.1)	22(75.9)	
N stage				0.001
N_0_	12	8(66.7)	4(33.3)	
N_1_	48	30(62.5)	18(37.5)	
N_2_	43	11(25.6)	32(74.4)	
N_3_	20	6(30.0)	14(70.0)	
M stege				0.376
M_1_	7	2(28.6)	5(71.4)	
M_0_	116	53(45.7)	63(54.3)	

### Western blotting

Cells at 70% to 80% confluence were washed 3 times in phosphate-buffered saline (PBS), and then lysed in SDS lysis buffer. Fresh tissue samples were ground to powder in liquid nitrogen and lysed with SDS-PAGE sample buffer. Equal amounts of protein samples (20 μg) were separated on 10.5% SDS polyacrylamide gels and transferred to PVDF membranes (Immobilon P, Millipore, Bedford, MA). Membranes were blocked with 5% fat-free milk in Tris-buffered saline containing 0.1% Tween-20 (TBST) for 1 h at room temperature. Membranes were incubated with 1:250-diluted anti-PTOV1 antibody (Sigma) overnight at 4°C, and then with horseradish peroxidase-conjugated goat anti-rabbit IgG (1:3,000). PTOV1 expression was detected using ECL prime Western blotting detection reagent (Amersham) according to the manufacturer’s instructions. Blotting membranes were stripped and reprobed with anti-GAPDH antibody (1:1,000; Sigma) as a loading control.

### Real-time PCR

Total RNA from cultured cells and fresh tissues were extracted using the Trizol reagent (Invitrogen) according to the manufacturer’s instruction. The extracted RNA was pretreated with RNase-free DNase, and 2μg RNA from each sample was used for cDNA synthesis with random hexamers. Real-time PCR primes were designed using the Primer Express Software Version 2.0 and the primer sequences are: *PTOV1* forward primer: 5'-CACCATCCCTCCATGTTGCTG-3'; *PTOV1* reverse primer: 5'-TCTTCATTGGCCTCATCCCC-3'; *GAPDH* forward prime: 5'-TGAACGGGAAGCTCACTGG-3'; *GAPDH* reverse primer: 5'-TCCACCACCCTGTTGCTGTA-3'. Expression data were normalized to the geometric mean of housekeeping gene GAPDH to control the variability in expression levels and calculated as 2^-[(Ct of PTOV1)–(Ct of GAPDH)]^, where Ct represents the threshold cycle for each transcript. All experiments were performed in triplicate.

### IHC analysis

Three of the freshly frozen noncancerous nasopharyngeal tissues were embedded with paraffin as normal controls. The immunohistochemistry was carried out as described previously [21]. The sections were incubated with a primary antibody to PTOV1 (Sigma) diluted at 1:100 overnight at 4°C. The degree of immunostaining was viewed and scored independently by two pathologists blinded to the clinical parameters. The scores were determined by combining the staining intensity and the proportion of positively stained tumor cells, giving rise to a Staining Index (SI) value for each sample. The staining intensity was graded according to the following criteria: 0 (no staining); 1 (weak staining = light yellow), 2 (moderate staining = yellow brown), and 3 (strong staining = brown). The proportion of tumor cells was scored as follows: 1 (<25% positive tumor cells), 2 (25–50% positive tumor cells), 3 (50–75% positive tumor cells) and 4(>75% positive tumor cells). The SI value was calculated as follows: SI = staining intensity × proportion of positively stained tumor cells. Scores given by the two independent investigators were averaged and evaluated comparatively for the expression of PTOV1 by SIs (scored as 0, 1, 2, 3, 4, 6, 8, 9 or 12). Cutoff values to define the high- and low-expression of PTOV1 were chosen on the basis of a measure of heterogeneity with the log-rank test statistics with respect to overall survival. An optimal cutoff value was identified, and the SI score of ≥6 was used to define tumors with high expression, and SI≤4 as tumors with low expression, of PTOV1. When comparative analysis of SIs was performed on a one-to-one basis, *t*-Test was used, and *P* < 0.05 was considered statistically significant.

IHC staining for protein expression in tumor lesions and normal tissues was quantitatively analyzed with the AxioVision Rel.4.6 computerized image analysis system assisted with an automatic measurement program (Carl Zeiss, Oberkochen, Germany). Specifically, the stained sections were evaluated at 200x magnification, and 10 representative staining fields of each section were analyzed to verify the Mean Optical Density (MOD), which represents the strength of staining signals as measured per positive pixels.

### Statistical analysis

All statistical analyses were carried out using the SPSS v. 16.0 statistical software packages. The relationship between PTOV1 expression and clinicopathological characteristics were analyzed by Chi-square test. The type of Cox regression model chosen was enter method. Survival curves were plotted by the Kaplan–Meier method and compared using the log-rank test. The following endpoints (measured from the date of diagnosis to the first defining event) were estimated: overall survival (OS) and progression-free survival (PFS). In all cases, a *P*-value of less than 0.05 was considered statistically significant.

## Results

### *PTOV1* is overexpressed in NPC cell lines

**Fig 1:**
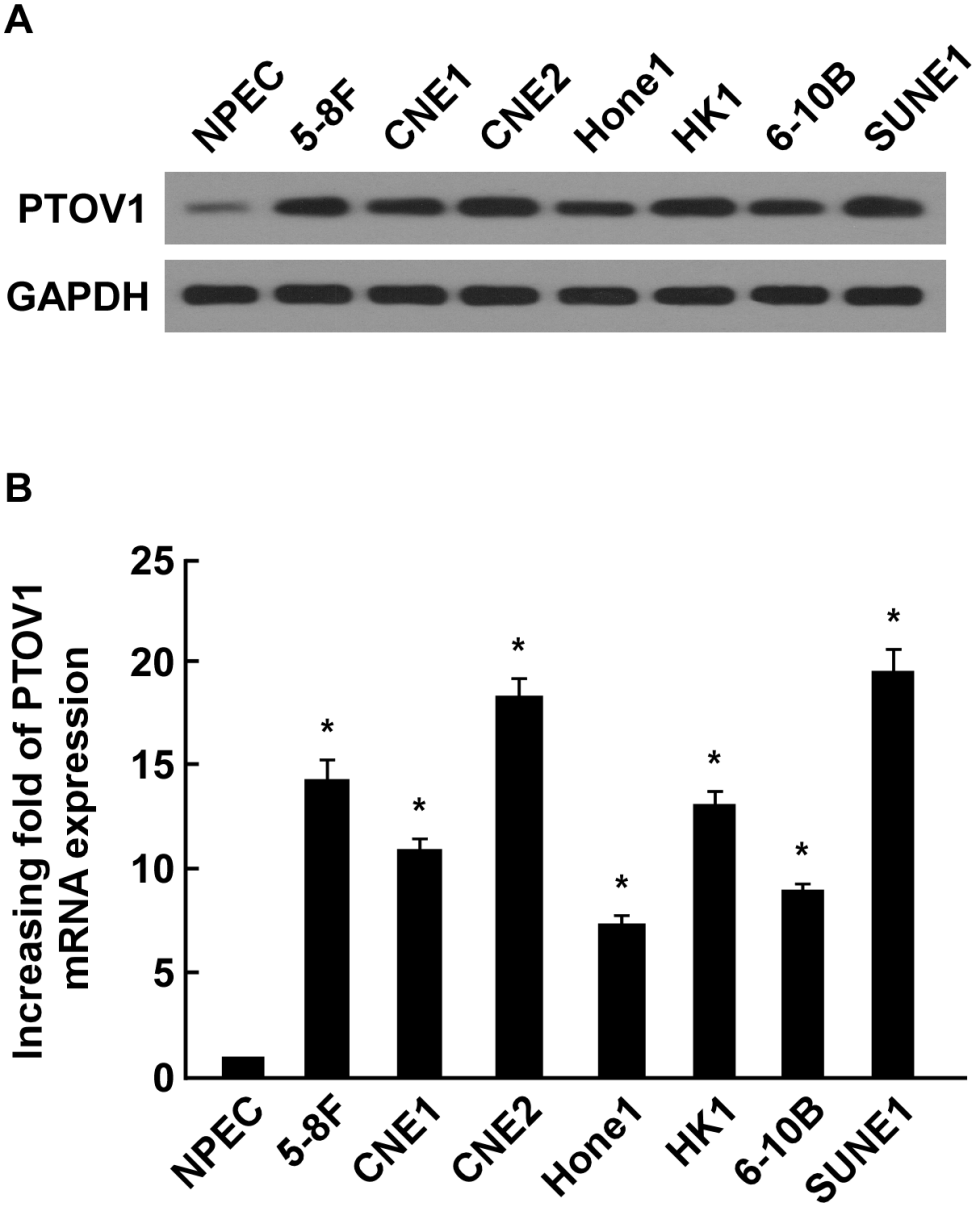
Overexpression of PTOV1 protein and mRNA in NPC cell lines. (**A** and **B**) Expression of PTOV1 protein and mRNA in NPC cell lines (5-8F, CNE1, CNE2, Hone1, HK1, 6-10B and SUNE1) and NPEC were examined by western blotting (**A**) and real-time PCR (**B**). Expression levels were normalized to GAPDH. Error bars represent the standard deviation of the mean (SD), calculated from three parallel experiments. * *P* < 0.05.

To evaluate the expression levels of PTOV1 protein and mRNA in NPC cell lines, western blotting and real-time PCR were performed. Western blotting analysis showed that PTOV1 was markedly overexpressed in all seven NPC cell lines, whereas it was weakly detected in Nasopharyngeal epithelial cells (NPECs) (Fig 1A). Real-time PCR was performed to detect and measure expression levels of PTOV1 mRNA. All seven NPC cell lines showed significantly higher expression levels of PTOV1 mRNA compared with NPECs (Fig 1B), which was consistent with the overexpression of PTOV1 protein measured in NPC cells. These results suggested that PTOV1 was upregulated in NPC cell lines.

### *PTOV1* is overexpressed in NPC tissues

**Fig 2:**
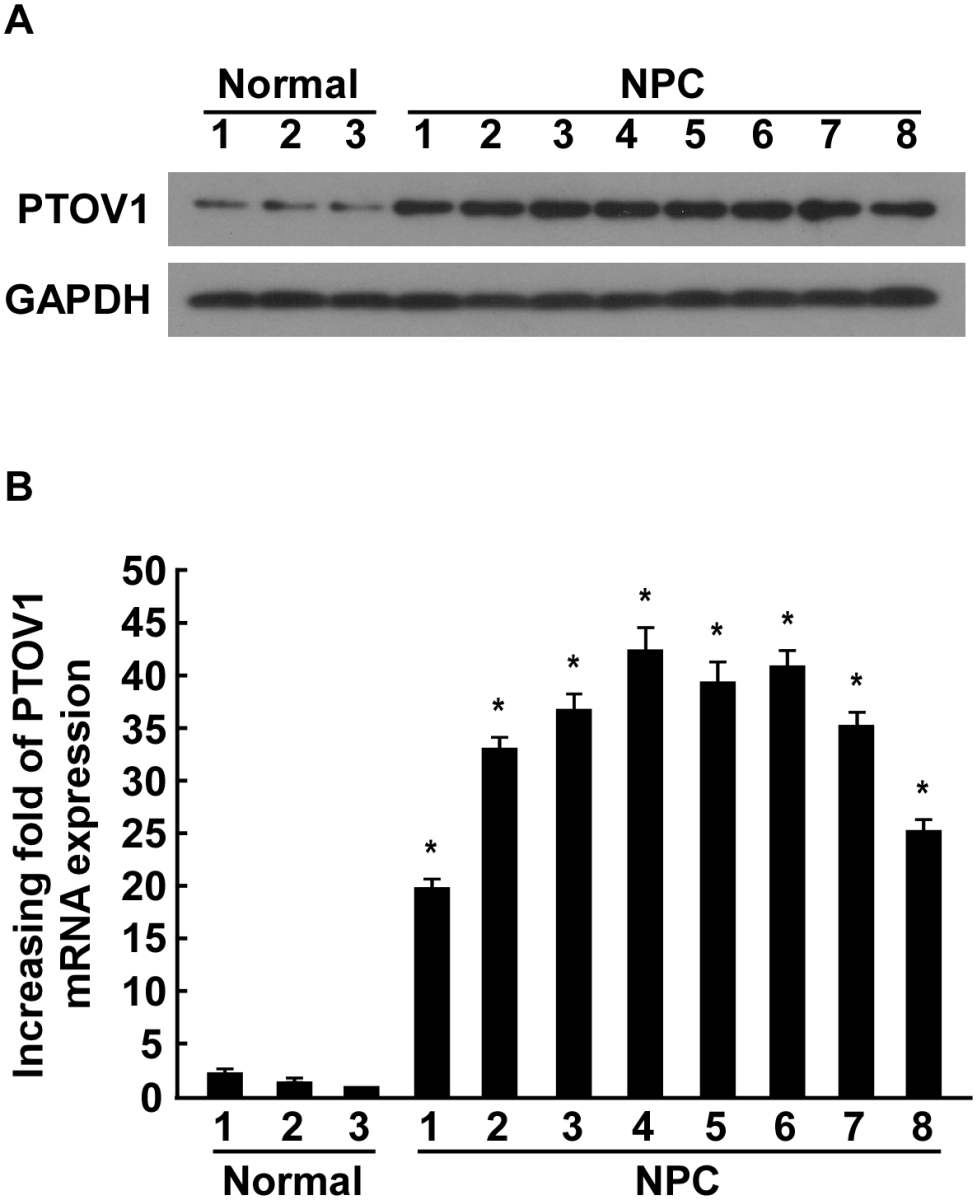
Overexpression of PTOV1 protein and mRNA in NPC tissues. (**A**) Expression of PTOV1 protein in three normal nasopharyngeal epithelial tissues and eight NPC tissues, measured by western blotting. GAPDH was used as a loading control. (**B**) Real-time PCR analysis of PTOV1 mRNA in three normal nasopharyngeal epithelial tissues and eight NPC tissues. Expression data were normalized to *GAPDH*. Error bars represent the SD, calculated from three parallel experiments. * *P* < 0.05.

As PTOV1 is overexpressed in NPC cell lines, we investigated PTOV1 expression in NPC biopsies. The expression of PTOV1 protein was overexpressed in all eight NPC samples, and was barely detectable in three normal nasopharyngeal epithelial tissue samples (Fig 2A). Consistently, real-time PCR analysis revealed that PTOV1 mRNA was upregulated in tumor samples (Fig 2B), further confirming that PTOV1 is overexpressed in NPC tissues.

**Fig 3:**
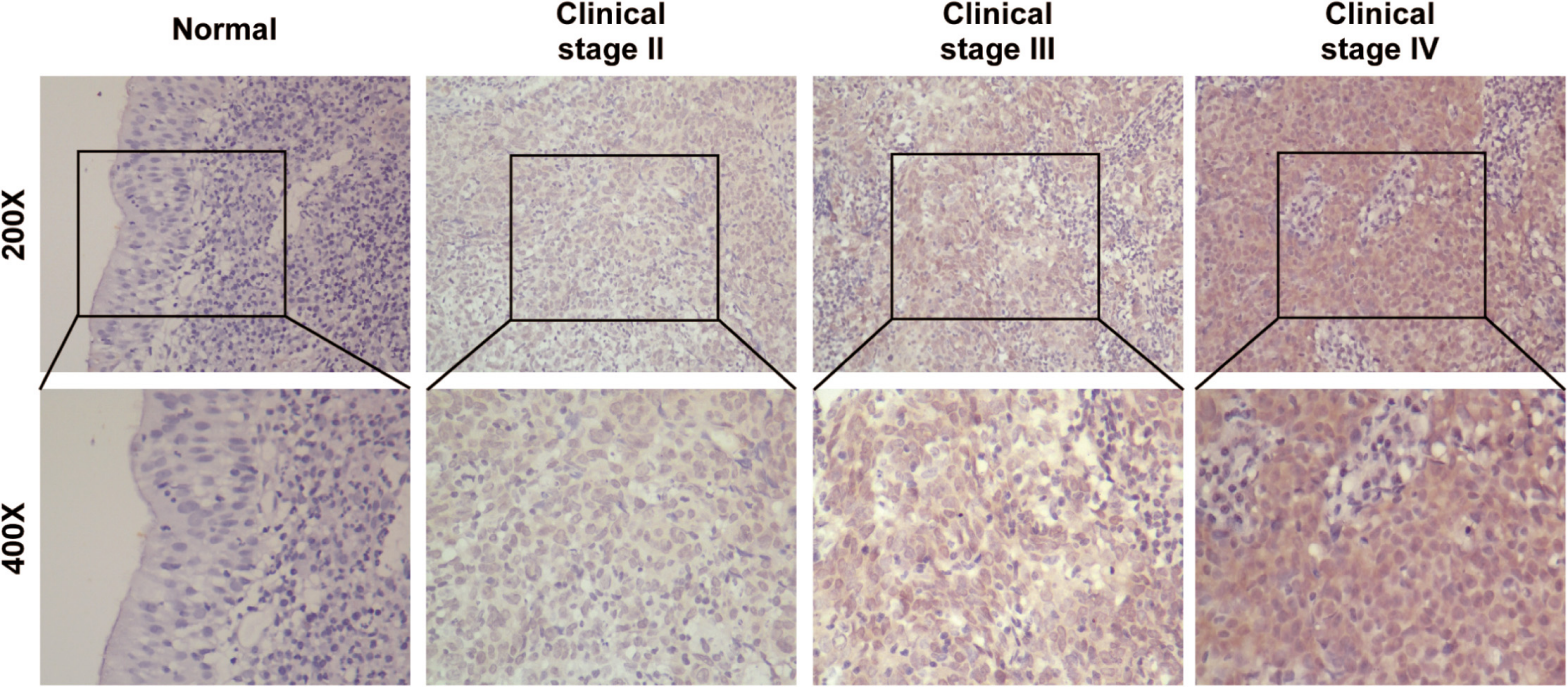
The expression of PTOV1 protein in archived NPC tissues from patients at different clinical stages.

To further demonstrate that PTOV1 protein is overexpressed in clinical samples of NPC, we performed immunohistochemistry (IHC) staining of paraffin-embedded archived biopsies (123 NPC samples and three normal nasopharyngeal epithelial samples). Consistent with the results above, no signals were detected in the normal nasopharyngeal epithelial tissues, while strong expression was observed in the tumor cells of NPC samples (Fig 3). Among 123 samples, high levels of PTOV1 expression were detected in 68 samples (55.3%) and weak or no staining was observed in 55 tumor samples (44.7%, Table 1). Expression of PTOV1 could be detected both in the nucleus and the cytoplasm of carcinomatous cells (Fig 3). Furthermore, Quantitative analysis revealed that the mean optical density (MOD) of PTOV1 staining in NPC samples increased with advancing clinical stage (P < 0.05, S1 Fig), indicating that PTOV1 expression in NPC increased with advancing clinical stage.

Taken together, PTOV1 mRNA and protein are upregulated in both NPC cell lines and NPC tissues, indicating a potential biological function of PTOV1 during NPC progression.

### *PTOV1* overexpression is associated with clinicopathological characteristics of NPC patients

To further investigate the possible correlations between PTOV1 expression levels and the clinicopathological characteristics of patients, statistical analyses were performed. Analysis of 123 NPC samples indicated that PTOV1 expression was markedly associated with clinical stage (*P* < 0.001), T classification (*P* = 0.042) and N classification (*P* = 0.001) (Table 1).

### Association between *PTOV1* expression and patients’ survival

**Fig 4:**
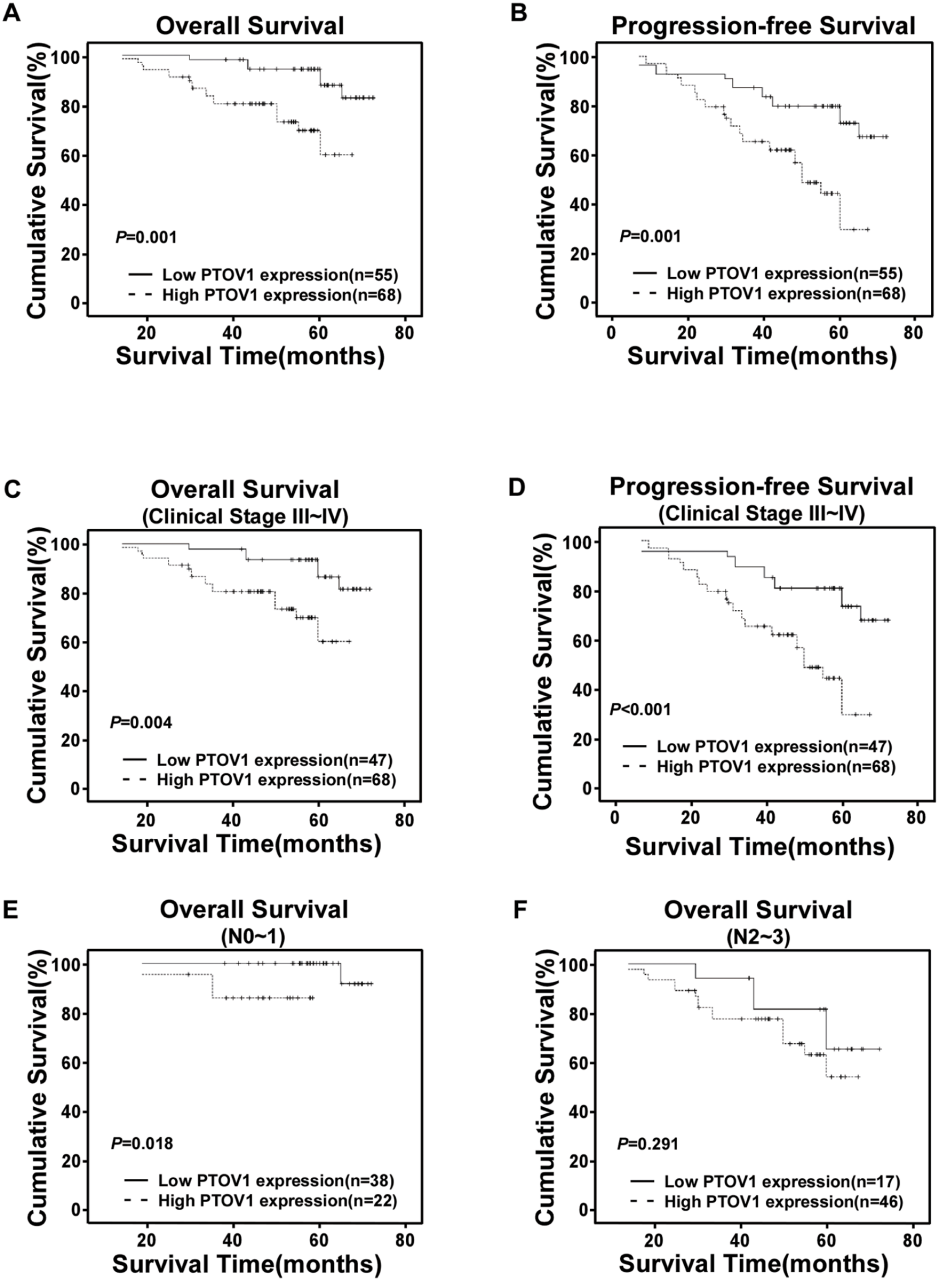
Kaplan-Meier curves with univariate analysis (log-rank). (**A** and **B**) OS (**A**) and PFS (**B**) rates for cases with high PTOV1 expression versus cases with low PTOV1 expression levels in all patients. (**C** and **D**) OS (**C**) and PFS (**D**) rates for late clinical stage cases (stage III/IV) with high PTOV1 expression versus cases with low PTOV1 expression levels. (**E**) OS rate for cases with high PTOV1 expression versus cases with low PTOV1 expression in patients with N0-N1 grade tumors. (**F**) OS rate for cases with high PTOV1 expression versus cases with low PTOV1 expression in patients with N2-N3 grade tumors.

Patient survival analysis showed an explicit negative correlation between the level of PTOV1 expression and both the overall survival (OS) and progression-free survival (PFS) of NPC patients (both *P* = 0.001, Fig 4A and 4B). The 5-year OS and PFS rates of the PTOV1 low expression group were 87.8% and 72.7%, respectively, whereas those of the PTOV1 high expression group were 59.6% and 29.4%, respectively.

The prognostic value of PTOV1 expression was analyzed in selected patient subgroups stratified according to clinical stage and N stage. Low levels of PTOV1 expression were observed in all the eight early stage (stage I–II) patient subgroups. As shown in Fig 4C and 4D, in the late stage (stage III-IV) subgroups, patients with higher PTOV1 expression level had significantly shorter OS and PFS durations compared with those with lower expression of PTOV1 (*P* = 0.004 and *P*< 0.001, respectively). However, when he data was stratified according to N stage, the impact on the outcome associated with overexpression of PTOV1 seemed to be more favorable only in the N0–1 subset (Fig 4E, log-rank test, *P* = 0.018), but not in the N2–3 subset (Fig 4F, log-rank test, *P* = 0.291).

### Univariate and multivariate analyses showed that *PTOV1* expression was a predictor of clinical outcomes

Univariate analysis indicated that in addition to elevated PTOV1 expression levels, N stage and M stage were significantly associated with worse survival for NPC patients (Table 2).

**Table 2:** Univariate analysis of prognostic factors for NPC patients.

Outcomes	Variable	B	p	HR	95%CI for HR
**OS**					
	**Sex (male vs. female)**	-0.197	0.640	0.821	0.359–1.876
	**Age (>44 vs. ≤44)**	-0.334	0.430	0.716	0.313–1.641
	**Clinical stage (IV vs. II&III)**	0.779	0.061	2.179	0.965–4.922
	**T stage (T3-4 vs. T1-2)**	-0.586	0.177	0.557	0.238–1.304
	**N stage (N2-3 vs. N0-1)**	1.725	0.002	5.614	1.915–16.457
	**M stage (M1 vs. M0)**	1.051	0.059	2.860	0.960–8.517
	**PTOV1 expression (high vs. low)**	1.499	0.003	4.475	1.668–12.010
**PFS**					
	**Sex (male vs. female)**	-0.118	0.7	0.889	0.488–1.619
	**Age (>44 vs. ≤44)**	-0.269	0.37	0.764	0.425–1.376
	**Clinical stage (IV vs. II&III)**	0.059	0.843	1.061	0.592–1.902
	**T stage (T3-4 vs. T1-2)**	0.063	0.86	1.065	0.528–2.149
	**N stage (N2-3 vs. N0-1)**	0.206	0.488	1.229	0.686–2.203
	**M stage (M1 vs. M0)**	1.046	0.018	2.845	1.196–6.768
	**PTOV1 expression (high vs. low)**	1.102	0.001	3.010	1.551–5.841

Abbreviations: CI = confident interval; HR = hazard ratio; OS = overall survival; PFS = progression-free survival

In a multivariate analysis, after adjusting for other risk factors, PTOV1 expression level was still an independent prognostic factor for OS (P = 0.03) and PFS (P<0.001). Higher levels of PTOV1 expression predicted worse OS (HR: 3.453; 95%CI: 1.13–10.545) and PFS (HR: 4.210; 95%CI: 1.900–9.327) (Table 3).

**Table 3:** Multivariate analysis of prognostic factors for NPC patients.

Outcomes	Variable	p	HR	95%CI for HR
**OS**				
	**Sex (male vs. female)**	0.437	0.703	0.289–1.710
	**Age (>44 vs. ≤44)**	0.509	0.745	0.311–1.785
	**Clinical stage (IV vs. II&III)**	0.276	1.688	0.658–4.335
	**T stage (T3-4 vs. T1-2)**	0.19	0.504	0.181–1.406
	**N stage (N2-3 vs. N0-1)**	0.087	2.842	0.858–9.414
	**M stage (M1 vs. M0)**	0.773	0.819	0.211–3.182
	**PTOV1 expression (high vs. low)**	0.03	3.453	1.13–10.545
**PFS**				
	**Sex (male vs. female)**	0.542	0.824	0.441–1.537
	**Age (>44 vs. ≤44)**	0.045	0.522	0.276–0.986
	**Clinical stage (IV vs. II&III)**	0.191	0.634	0.320–1.254
	**T stage (T3-4 vs. T1-2)**	0.244	1.785	0.673–4.731
	**N stage (N2-3 vs. N0-1)**	0.223	0.635	0.305–1.319
	**M stage (M1 vs. M0)**	0.002	8.18	2.209–30.282
	**PTOV1 expression (high vs. low)**	<0.001	4.210	1.900–9.327

Abbreviations: CI = confident interval; HR = hazard ratio; OS = overall survival; PFS = progression-free survival

In conclusion, the results of this study suggested that the expression of PTOV1 could be considered as an independent and valuable prognostic marker for prediction of NPC patients’ outcomes.

## Discussion

In this report, we demonstrated, for the first time, the clinical significance of PTOV1 overexpression in NPC patients. Our results clearly showed PTOV1 mRNA and protein levels are upregulated in NPC cell lines and NPC specimens, in comparison with noncancerous nasopharyngeal epithelial cells and tissues. IHC analyses indicated that the overexpression of the PTOV1 protein correlates with unfavorable prognosis of NPC patients, especially in lower N stage subsets. Multivariate Cox regression analyses suggested that PTOV1 was an independent prognostic indicator for survival of NPC patients. Taken together, our study suggested that PTOV1 might represent a novel biomarker for NPC diagnosis and prognosis.

PTOV1 was initially implicated in stimulation of prostate carcinoma cells’ proliferation, and its overexpression was reported to be a more general feature associated with neoplastic cells, especially in human high-grade malignant tumors [8–10, 14]. This gene has a mitogenic function that shuttles between the nucleus and the cytoplasm in a cell cycle-dependent manner in PCa cells [8–10]. In addition, PTOV1 overexpression facilitated entry of PCa cells into the S phase of the cell division cycle [1, 15, 16]. Thus, these findings indicated that the overexpression of PTOV1 might contribute to NPC development and progression. However, our findings confirmed that PTOV1 is overexpressed in nasopharyngeal carcinoma and may act as a novel predictor for prognosis and survival of NPC patients. Nevertheless, further investigations are required to determine the molecular mechanisms of PTOV1’s involvement in the development and progression of NPC.

Our present study also demonstrated that PTOV1 was overexpressed in 68 NPC samples (55.3%) and its expression correlated with survival time in all 123 NPC patients. Moreover, we investigated its role in selective subgroups stratified based on clinical stage and N stage. Similar to the results shown by Fernández S *et al*., that PTOV1 expression was significantly associated to tumor progression [14], we found that increased expression of PTOV1 correlated with a higher degree of tumor stage and reduced survival time of NPC patients, especially in the late stage (stage III-IV) patient subsets. However, considering that more than 90% of the analyzed NPC tissues were of stage III/IV in our study, we cannot exclude the possibility that PTOV1 expression correlates with survival time of the patients at an early clinical stage. Multivariate analyses revealed that both PTOV1 expression level and N classification are independent prognostic factors. Specifically, we observed that the prognostic potential of PTOV1 expression was only found in N0-1 patient’s subsets, but not in the N2–3 subsets. This finding illustrated that PTOV1 may play an important role in the initial phase of NPC lymph node metastasis. Besides, studies have shown that NPC patients with a higher N stage have poorer treatment outcomes compared with patients without lymph node metastasis or with lower N stage nodal metastasis [22]. Thus, the upregulation of PTOV1 could aid in detecting early lymph node metastasis of NPC patients and guiding their follow-up schedule.

The chromosomal region containing PTOV1, 19q13.3 ± 13.4, harbors various genes relevant to cell proliferation and mitogenicity enhancement. Many of these genes and their encoded proteins are involved in carcinogenesis and are considered novel potential cancer biomarkers, such as the proteases prostate specific antigen [23], kallikrein-related peptidase 6 (KLK6) [24], or the apoptosis regulator bax-δ [25]. These previous observations inspired us to explore the biological role of PTOV1 in NPC. Our data confirm that PTOV1 might be a new oncogenic factor associated with poor prognosis in NPC.

Recent publications reported that PTOV1 cooperates with Zyxin in retinoic acid receptor (RAR) repression by abolishing the binding of the RAR coactivator CBP to the RAR target in non-small-cell lung cancer cells [16, 17]. Retinoic acid (RA) can inhibit the growth of the nasopharyngeal carcinoma cell line HNE [26]. Many reports have observed the chemically-induced behavior of nasopharyngeal carcinoma cells; therefore, drug resistance regulated by PTOV1 represents attractive target for NPC therapy. However, further studies are required to determine the detailed mechanism of PTOV1-associated signaling regulation in NPC development.

In conclusion, this is the first study to evaluate PTOV1 as a novel biomarker for disease diagnosis and prognosis of patient survival outcomes in NPC. We also suggest that determining PTOV1 expression levels may help to detect early lymph node metastasis of NPC patients, permitting the stratification of patients for the selection of treatments. To this end, further investigation on the molecular mechanism of PTOV1’s association with the development and progression of NPC and prospective studies on the prognostic significance of PTOV1 are eagerly awaited.

## Conclusions

In this study, we demonstrated that PTOV1 overexpression is associated with poor survival outcomes of NPC patients, especially in N0-1 patients. Multivariate analyses revealed that PTOV1 expression level could be an independent prognostic marker for survival in NPC patients. Therefore, testing the PTOV1 protein level may help to detect early lymph node metastasis of NPC patients and serve as an independent prognostic biomarker for human NPC.

## Supporting Information

S1 Dataset Original data used for all statistical analyses in this article. PTOV1 expression: 1(SI score of ≤4 was used to define tumors with low expression of PTOV1) and 2(SI≥6 as tumors with high expression of PTOV1). Status: 0(alive) and 1(death).
(XLSX)

S1 Fig Statistical quantification of the average MOD of PTOV1 staining between normal nasopharyngeal epithelial tissues and NPC samples of different clinical stages. PTOV1 expression gradually increased from Stage II though to Stage IV. PTOV1 expression was undetectable in normal nasopharyngeal epithelial tissues, marginal in Stage II, moderate in Stage III, and strong in Stage IV. *P <0.05 (compared with normal nasopharyngeal epithelial tissues).
(TIF)
