# Supplementary figures and images for: Prostate Tumor Overexpressed 1 (PTOV1) Is a Novel Prognostic Marker for Nasopharyngeal Carcinoma Progression and Poor Survival Outcomes
Source: PLoS One. 2015 Aug 25;10(8):e0136448. doi: 10.1371/journal.pone.0136448 (PMC4549246; doi:10.1371/journal.pone.0136448)

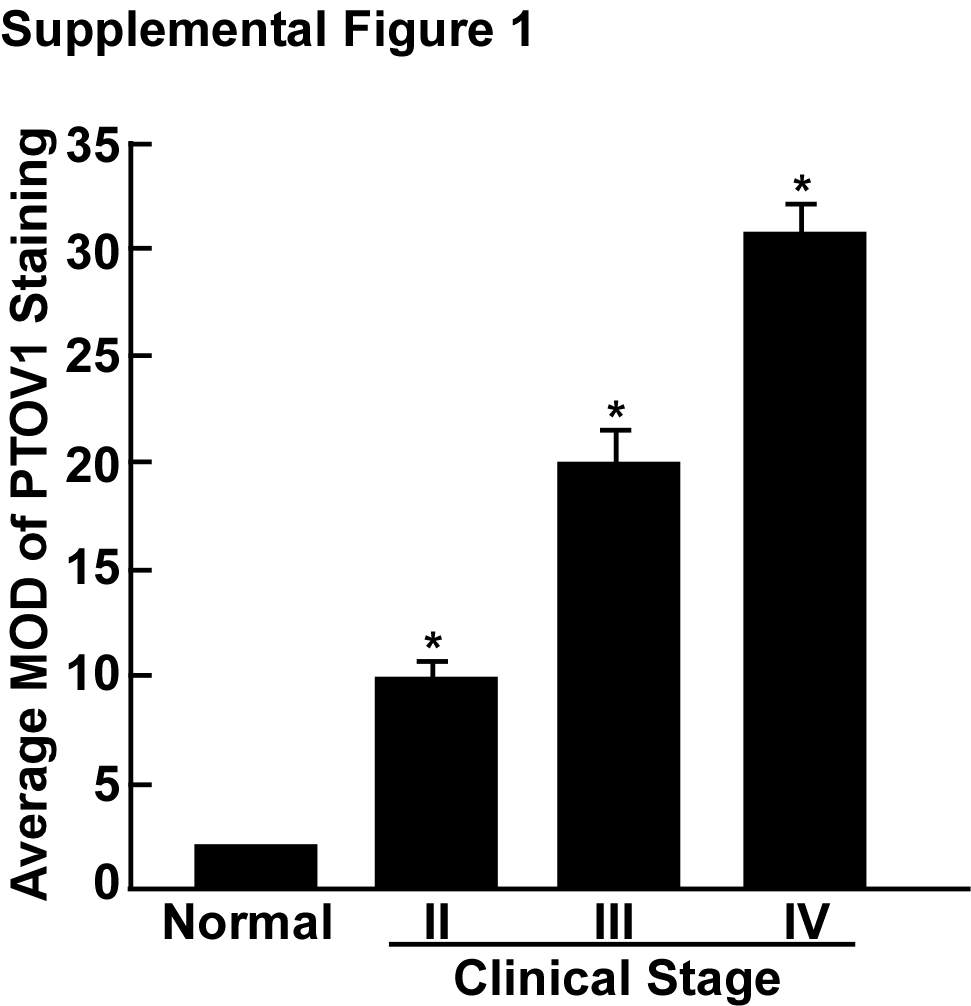

Supplement: S1 Fig — PTOV1 expression gradually increased from Stage II though to Stage IV. PTOV1 expression was undetectable in normal nasopharyngeal epithelial tissues, marginal in Stage II, moderate in Stage III, and strong in Stage IV. *P <0.05 (compared with normal nasopharyngeal epithelial tissues). (TIF) [file pone.0136448.s002.tif]
